# Budding baboon blues: the prenatal diet lands a lasting gut punch

**DOI:** 10.1152/ajpgi.00252.2025

**Published:** 2025-08-20

**Authors:** Anna Grigorian, Mengyun Wu, Jerrold R. Turner

**Affiliations:** Laboratory of Mucosal Pathobiology, Department of Pathology, Brigham and Women’s Hospital and Harvard Medical School, Boston, Massachusetts, United States

Multiple maternal and neonatal factors have been associated with increased risk of gastrointestinal and systemic disease in newborns. Those confirmed in clinical studies include poor maternal nutrition, maternal substance abuse, chorioamnionitis, and placental insufficiency (1, 2) as well as low neonatal birth weight and prematurity (3, 4). Preclinical studies have provided additional data suggesting that an immature gut microbiome, epithelial functional defects, intestinal barrier loss, stress, bacterial translocation, or TLR4 activation may also contribute to the development of postnatal diseases, such as necrotizing enterocolitis, in the newborn (5–10). Most preclinical studies have focused on rodent models, with only a few making use of nonhuman primates despite their greater relevance to human biology (1, 11–16). In this journal, Gershner et al. (17) advance this work by studying the consequences of a maternal high-carbohydrate, high-fat, Western-like diet using nonhuman primates (Fig. 1).

Gershner et al. (17) sought to explore the potential relationship between diet and outcome using female baboons that were fed a conventional baboon diet or a high-carbohydrate, high-fat, Western-like diet (WD) before mating. Previous work using the same model showed that feeding WD to females before conception was sufficient to modify the gut microbiome, increase HDL, LDL/VLDL, and serum inflammatory markers, and induce metabolic syndrome and steatohepatitis relative to a females fed a conventional baboon diet (12, 15). Dams on WD displayed altered placental RNAi expression and enhanced placental macrophage infiltration (12). These results parallel those of similar studies using mice (9).

Previous analyses in nonhuman primates have shown that maternal WD during pregnancy and lactation modifies the postweaning juvenile microbiome (15). In the present study, Gershner et al. (17) eliminated potential effects of both the maternal vaginal microbiome and the offspring’s microbiome by studying fetuses delivered via C-section at 90% gestation. TLR4, IL-8, and IFN-γ transcript numbers were increased within the ileal tissue of fetuses born to dams that had been fed a WD for 1 year before conception. In contrast, ileal cytokine transcripts of fetuses born to dams that received WD for 3 mo before mating were not different from those of fetuses born to conventional diet-fed dams. Although the reason for this difference is not clear, the impact of a longer WD exposure is consistent with a report that 2 years of WD are required to induce weight gain in nonhuman primates (16). This time-dependent effect of WD exposure may also explain differences between these data and published studies with preconception WD exposures from 0 to 8 years (11, 14–16).

In 2–3-yr-old juveniles, maternal WD was associated with increased IFN-γ and IL-6 transcripts within the ileum. In contrast to fetuses, neither TLR4 nor IL-8 was altered in juveniles. Several other cytokines examined, including TNF and IL-1β, were also similar in juveniles regardless of maternal diet.

The persistence of maternal WD effects in juveniles 2 years after weaning onto a conventional diet is, perhaps, the most remarkable finding in this study. Previous non-human primate studies, found that the effects of maternal WD were strongest in juveniles that also received WD. Here, Gershner et al. (17) continued the dams on WD but weaned all baboons, regardless of maternal diet, onto a conventional diet. Thus, we can conclude that, at least in baboons, the effects of maternal diet persist into adolescence. One caveat is that there may have been indirect WD exposure after birth, as the dams were maintained on either a WD or conventional diet while they were nursing. However, because baboons are weaned at 12–18 mo of age, the juveniles were not actively exposed to WD at the time of analysis. Alternatively, the data could reflect differences in the neonatal gut microbiome, which is derived from the maternal vaginal microbiome during birth. This idea is supported by previous work from this group showing that WD modifies the maternal gut microbiome (12). Future studies with longitudinal assessments of maternal, infant, and juvenile microbiota could provide new insight into the mechanisms by which WD-induced changes are transmitted to offspring.

Of course, potential effects of maternal diet on the epithelium, whether mediated by microbial, immune, or other changes, must also be considered. There was a modest, qualitative decrease in villous epithelial ZO-1 expression in fetuses, but not juveniles, of WD-fed dams. Even in the case of the fetuses, this result is difficult to interpret given previous work showing that intestinal epithelial ZO-1 (*Tjp1*) knockout mice do not develop spontaneous disease and are able to maintain the gut barrier (18).

Given the technical challenges of functional analyses in the fetal and juvenile baboons, Gershner et al. (17) turned to ileal epithelial stem cells culture using methods similar to those used to culture human enteroids. Barrier function, measured as transepithelial electrical resistance, of enteroid-derived monolayers from a fetus of WD-fed dams was less than half that of monolayers derived from a fetus of dams fed a control diet. Moreover, LPS and hypoxia induced greater production of IL-8 and TNF by epithelial cells derived from the WD fetus relative to those from the conventional diet fetus. These data are intriguing but inconclusive given that they are based on enteroids derived from one fetus for each maternal diet. Further study, including more comprehensive quantitative analyses of tight junction and other proteins and, possibly, ex vivo functional analysis of barrier function using Ussing chambers, may shed light on these observations.

Despite these weaknesses, the data are of great interest, particularly because the studies were performed in nonhuman primates. The results add to existing work in nonhuman primates and mice linking maternal diet to pregnancy outcomes. However, similar studies in human subjects have generally failed to detect any differences between neonates based on maternal diet.

Arguably, the most pressing unanswered question remains: what is the effect of maternal diet on pregnancy outcomes? One could also ask if diet impacts postnatal growth, alters sensitivity to exogenous stimuli, or modifies the risk of developing specific disorders. While these questions remain, the limited data reinforce what might have been anticipated: that a maternal WD rich in carbohydrates and fats is not beneficial, at least in baboons and mice. Although one could speculate that this extends to humans, rigorous analyses are not available. Thus, as is often the case in medical practice, we must make recommendations without evidence-based data. In keeping with the principle of primum non nocere—first, do no harm—it seems reasonable to consider that a balanced maternal diet may be preferable to a typical Western diet, as demonstrated in other aspects of health and nutrition.

## Figures and Tables

**Figure 1.**
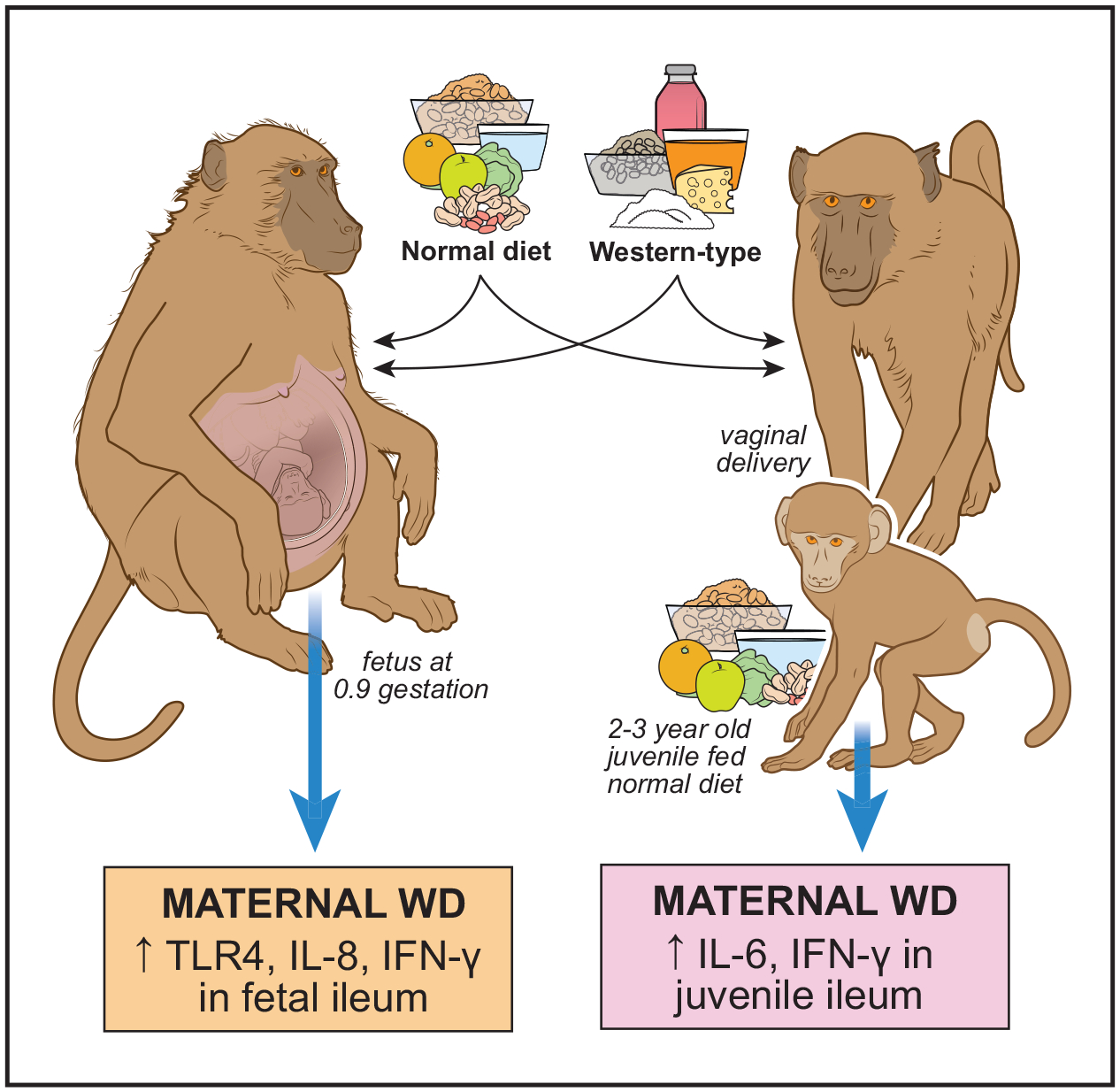
Gershner et al. (17) analyzed the effects of a maternal high-carbohydrate, high-fat Western-like diet on pregnancy outcomes. Female baboons were fed either a conventional baboon diet or a high-carbohydrate, high-fat Western-like diet (WD) before mating. Ileal samples were analyzed from fetuses delivered by C-section at 90% of gestational age and from vaginally born 2–3-yr-old juveniles, all of whom received the control diet at weaning.
